# Effects of Dietary L-TRP on Immunity, Antioxidant Capacity and Intestinal Microbiota of the Chinese Mitten Crab (*Eriocheir Sinensis*) in Pond Culture

**DOI:** 10.3390/metabo13010001

**Published:** 2022-12-20

**Authors:** Mengna Hou, Yangyang Pang, Chao Niu, Dongxin Zhang, Ying Zhang, Zhiqiang Liu, Yameng Song, Aoya Shi, Qing Chen, Junyan Zhang, Yongxu Cheng, Xiaozhen Yang

**Affiliations:** 1 National Demonstration Center for Experimental Fisheries Science Education, Shanghai Ocean University, Shanghai 201306, China; 2 Key Laboratory of Freshwater Aquatic Genetic Resources, Ministry of Agriculture, Shanghai Ocean University, Shanghai 201306, China; 3 Engineering Research Center of Aquaculture, Shanghai Ocean University, Shanghai 201306, China

**Keywords:** *Eriocheir sinensis*, L-tryptophan, immunity, antioxidant capacity, intestinal microbiota

## Abstract

L-tryptophan (L-TRP) is an essential amino acid for the normal growth of crustaceans. As a nutritional supplement and antioxidant, L-TRP has the function of immune and antioxidant capacity regulation. From July to November, the effects of L-TRP on the immunity, antioxidant capacity and intestinal microflora of the Chinese mitten crab (*Eriocheir sinensis*) in pond culture were investigated. After feeding an L-TRP diet for 30 (named as August), 60 (named as September) and 106 (named as November) days, respectively, the activities of the immune and antioxidant enzymes in the hepatopancreas and hemolymph were evaluated, and the intestinal microbiota were profiled via high-throughput Illumina sequencing. The results showed that supplementation of L-TRP significantly increased the activities of AKP in the hepatopancreas in September, and significantly increased the activities of ACP in the hepatopancreas in August and September, and the hemolymph’s ACP activities also significantly increased in August and November (*p* < 0.05). Similarly, the activities of SOD, AOC and POD in the hepatopancreas significantly increased in September and November (*p* < 0.05) after feeding the L-TRP diet; meanwhile, the activities of SOD and AOC in the hemolymph also significantly increased in August (*p* < 0.05). However, in August, the L-TRP diet resulted in a significant increase in MDA activity in the hepatopancreas and hemolymph (*p* < 0.05). In addition, the results of the intestinal microbiota analysis showed that Firmicutes, Bacteroidetes and Proteobacteria were the dominant phyla in August, September and November, and Patescibacteria was the dominant phylum in September and November. After feeding the L-TRP diet, the richness of *Cyanobacteria* and *Desulfobacterota* significantly increased in August (*p* < 0.05), and the richness of *Actinobacteriota* significantly decreased in September (*p* < 0.05). Moreover, the L-TRP supplementation significantly reduced the abundance of ZOR0006 in the Firmicutes in September (*p* < 0.05). In conclusion, dietary L-TRP could improve the immunity and antioxidant ability and impact the intestinal health of *E. sinensis* at the early stage of pond culturing. However, long-term feeding of an L-TRP diet might have no positive impact on the activities of the immune, antioxidant enzymes and intestinal microbiota.

## 1. Introduction

The Chinese mitten crab (*Eriocheir sinensis*) is an economically important crab in China. It is loved by consumers because of its delicious taste and high nutritional value. In recent years, the rapid development of pond culture of *E. sinensis* has also brought a great challenge to the healthy growth and survival of crabs. Previous studies show that crustaceans are sensitive to virus and bacterial infections, due to improper dietary nutritional supply and environmental management [1]. In the process of crab pond culture, the pathogenic bacteria [2], viruses [3] and parasites [4], which erupt frequently, can reduce their immunity and affect the survival rate of the animals. Previously, a series of immunopotentiators was proposed to reduce the damage of bacterium and virus infections to aquatic animals. However, these immune enhancers are not enough to give crustaceans adequate protection. Therefore, identifying powerful immunity-enhancing feed additives is particularly critical for the healthy breeding of crustaceans.

L-tryptophan (L-TRP) is one of the eight essential amino acids necessary for humans and animals. It shares a variety of physiological functions, including improvement in body immunity, anti-oxidation and other regulatory functions [5,6]. However, L-TRP is limited synthetically and only ingested through food in aquatic species [7]. Dietary L-TRP deficiency can reduce the levels of nutrition and suppress the immune function, which leads to a significant increase in the susceptibility to disease and an increase in the morbidity and mortality of European seabass (*Dicentrarchus labrax*) [8]. On the other hand, TRP supplementation was able to promote the immune status and disease resistance of Senegalese sole (*Solea senegalensis*) [9]. Non-specific immunity is the principal immune defense system in crustaceans, including a variety of endogenous expressions, induction factors and immune enzymes. Among these immune response substances, alkaline phosphatase (AKP) and acid phosphatase (ACP), as specific indicators, are usually used to evaluate the non-specific immune status of crustaceans [10]. A study has shown that AKP plays an important role in absorbing and utilizing the nutrients in aquatic animals and contributes to resist disease, whereas ACP plays an important role in immune response [10]. Generally, AKP and ACP are composed of a variety of phosphomonoesterases and are very important defense enzymes in the immune system of crustaceans [11]. Furthermore, our previous study confirmed that dietary L-TRP can significantly increase the activity of AKP in the hemolymph and has a specific role in promoting the nonspecific immunity of *E. sinensis* in a short-term laboratory experiment [12]. Therefore, we hoped to further evaluate the effect of L-TRP supplementation on the ACP and AKP activities in actual pond culturing.

Antioxidant capacity is another important indicator and reflects immunocompetence and the healthy condition of animals [13]. The total antioxidant capacity (T-AOC), superoxide dismutase (SOD), peroxidase (POD) and malondialdehyde (MDA) are usually used to characterize antioxidant capacity [14,15,16]. L-TRP, as an antioxidant, can also impact aquatic animals’ antioxidant capacity. For example, L-TRP supplementation improves the antioxidant capacity of fish and enhances the activity of the major antioxidant enzymes [17]. Moreover, another study has shown that an optimal dietary L-TRP level can improve the SOD and AOC activities of *Megalobrama amblycephala*, indicating that it has protective effects on oxidative damage [18]. In addition, the MDA content in rainbow trout was significantly decreased after feeding a 0.5% TRP diet, and the antioxidant performance of rainbow trout was improved [19]. The same results have been found in hybrid catfish, *Pelteobagrus vachelli* ♀ × *Leiocassis longirostris* ♂ [20]. However, long-term L-TRP supplementation has a negative impact on the overall welfare status of pikeperch (*Sander lucioperca*) [21]. Nevertheless, the effect of long-term feeding of a L-TRP diet in pond culture on the immune and antioxidant capacity of *E. sinensis* is unclear and needs further exploration.

In addition, animals’ intestines contain a large number of microbiota that host microorganisms. Under normal conditions, the microorganisms will maintain a dynamic balance with their host and regulate the digestion, metabolism, nutrient absorption, energy supply and immune response of the host [22]. However, many factors, including the bottom mud of the pond, aquatic water, temperature, residual bait, and also diseases and diet composition, can affect the homeostasis of the intestinal microbiota of aquatic animals in pond culture and lead to a decline in physical health. A previous study has demonstrated that L-TRP can improve intestinal microbiota [23]. In addition, another research study also suggested that dietary TRP can improve the intestinal microbiota and regulate nutrient transport and metabolism in triploid crucian carp [24]. In fact, our previous study confirmed that feeding L-TRP in the short term can significantly increase the richness and diversity of intestinal microbiota in *E. sinensis* [12]. Therefore, L-TRP is a potential feed additive for regulating intestinal microbiota. However, there has also been a lack of research to explore the effect of L-TRP on the intestinal microbiota of *E. sinensis* in actual pond culture.

L-TRP should be comprehensively evaluated as a potential immune enhancer. There is a great difference between a laboratory short-term experiment and the long-term actual pond culturing environment. In this study, we mainly investigated the effects of dietary supplementation of L-TRP on immune response, antioxidant capacity and intestinal microbiota of *E. sinensis* in pond culture to provide pieces of evidence for the practical application of L-TRP.

## 2. Materials and Methods

### 2.1. Experimental Diets

The commercial pellet feed, which was designed for *E. sinensis*, was purchased from Anhui Huayi Co., Ltd. (Hefei, China). The L-TRP (specification for biological reagent, 25 g, measured concentration ≥ 99.7%) was purchased from Sinopharm Chemical Reagent Co., Ltd. (Shanghai, China). According to previous studies [25,26], the pellet feed was fully crushed by a superfine crusher (800A, Baihaojia, Shenzhen Elsa Technology Co., Ltd., Shenzhen, China); the size of the powdered feed should be less than 60 mesh. Then, 0.75% L-TRP was added using a stepwise enlargement method and mixed evenly. After that, the mixture was added to 40% water. A 4 mm diameter pellet feed was prepared by a double-screw extruder. Finally, the pellet feed was dried at 55 °C in an oven and cooled under natural conditions. The pellet feed was sealed in zip lock bags and stored in a refrigerator at −20 °C until use. The actual content of L-TRP in the diets was detected by the testing center of Shanghai Jiaotong University. The final crude protein content of the feed was 38.26% in the control group and 39.85% in the L-TRP group. The actual content of L-TRP in the control group and the L-TRP group was 0.393% and 0.949%, respectively.

### 2.2. Experimental Design and Animal Culturing

The male *E. sinensis* were purchased from Chongming Island (the experimental base of Shanghai Ocean University). The animal study protocols were approved by the Animal Research and Ethics Committee of Shanghai Ocean University (approval ID: SHOU-DW-2021-084; approval date: 10 March 2021). The intact and healthy crabs, with a body weight of 27.15 ± 3.49 g, were equally divided into eight identical cages (length: width: height = 6 m:5 m:2 m; area: 30 m^2^), which were constructed in a pond with a water depth of 1–1.2 m. The cages were divided into the control group (four cages) and the L-TRP group (four cages). Initially 40 crabs were stocked in each cage. After three days, we replenished with new crabs to reach 40, according to the number of dead observed in each cage. Polyethylene anti-escape film with a width of 20 cm was installed on the edge of each cage, and two microporous aeration plates were placed on the bottom of each cage in order to increase the dissolved oxygen. A total of 16 microporous aeration plates were connected in a series to a 2.2 kW aerator. After one week of temporary feeding, the animals were fed the experimental diets. The crabs in each cage were fed an experimental diet according to 2–3% of the total crab biomass, once daily from 17:30 to 18:30. The next day, the feed amount of each cage was adjusted according to the residual feed in the disc-shaped net. The DO and water temperature were recorded every day (7:00 or 18:00) using a handheld multiparameter meter (HQ30d, HACH, Loveland, CO, USA), and the pH was detected by a handheld pH meter (AS600, As One Corporation, Osaka, Japan). The commercial detection kits (SOUTH RANCH, Zhengzhou, Henan, China) were used once a week to determine the concentrations of water, ammonia and nitrite. During the experimental period, the main water parameters were as follows: natural light cycle; the temperature was maintained at 15–32 °C; DO > 4 mg/L; ammonia < 0.3 mg/L; nitrite < 0.10 mg/L; and the pH was 8–8.5.

### 2.3. Sample Collection

The feeding experiment lasted for a total of 106 days (28 June–12 November), and sampling was carried out on the 30th, 60th and 106th days from the beginning of the formal feed experiment. At every sample point, three crabs were randomly captured and dissected from each cage. A total of 12 crabs from each group were used to collect the hepatopancreas, hemolymph and intestinal contents. The crabs were put on ice for anesthetic treatment before the sampling. After dissecting, all the tissues were immediately snap-frozen in liquid nitrogen and then stored at −80 °C until use. In addition, the intestinal contents were handled under sterile conditions. The hemolymph samples were collected from the third limb of each crab with a disposable sterile syringe.

### 2.4. Analysis of Immune and Antioxidant Capacity

In this study, the immune response and antioxidant capacity of hepatopancreas and hemolymph were analyzed with several commercial kits, which were purchased from the Nanjing Jiancheng Institute of Biological Engineering (Nanjing, China). According to the operating instructions, the hemolymph samples were homogenized with a homogenizer and centrifuged at 42,000× *g* for 10 min at 4 °C, and then the centrifuged hemolymph supernatant was taken. An amount of 0.3 g of the hepatopancreas from each crab was weighed and placed into a 5 mL centrifuge tube. Next, the crabs’ physiological saline solution was added into the tube at a ratio of 1:9 (*w*/*v*) [27] and then was homogenized using a homogenizer (T 10 basic ULTRA-TURRAX^®^, IKA). Subsequently, these tissue homogenates were centrifuged at 42,000× *g* at 4 °C for 10 min. After that, the supernatant was taken and determined by a UV-spectrophotometer (T6 New Century Beijing Purkinje General Instrument Co., Ltd., Beijing, China). According to the manufacturer’s guidelines, the alkaline phosphatase (AKP) and phosphatase (ACP) contents were determined using the UV-spectrophotometer at 520 nm for the absorbance values, and the total antioxidant capacity (T-AOC), total superoxide dismutase (SOD), peroxidase (POD) and malondialdehyde (MDA) were determined at 520 nm, 550 nm, 420 nm and 532 nm, respectively.

### 2.5. Analysis of Intestinal Microbiota

#### 2.5.1. Total DNA Extraction, 16S rRNA Amplification, Purification and Quantification

In this experiment, a total of 36 samples were used to extract the DNA (*N* = 6). The total microbial community DNA was extracted from the feces samples using the E.Z.N.A. soil DNA Kit (Omega Bio-tek, Norcross, GA, USA), according to the manufacturer’s instructions. The DNA extraction was checked using a 1% agarose gel, and the DNA concentration and purity were determined by a NanoDrop2000 UV-Vis spectrophotometer (Thermo Scientific, Wilmington, NC, USA). The hypervariable region V3–V4 of the 16S rRNA gene was amplified with the primer pairs 338F (5′-ACTCCTACGGGAGGCAGCAG-3′) and 806R (5′-GGACTACHVGGGTWTCTAAT-3′). The amplification procedure was as follows: initial denaturation at 95 °C for 3 min, followed by 27 cycles of denaturing at 95 °C for 30 s, annealing at 55 °C for 30 s and extension at 72 °C for 45 s, then single extension at 72 °C for 10 min, and finally ending at 4 °C (PCR instrument: ABI GeneAmp 9700). The PCR mixture contained 5 × TransStart FastPfu buffer 4 μL, 2.5 mM dNTPs 2 μL, forward primer (5 μM) 0.8 μL, reverse primer (5 μM) 0.8 μL, TransStart FastPfu DNA Polymerase 0.4 μL, template DNA 10 ng·μL^−1^ and, finally, ddH2O up to 20 μL. The PCR reactions were performed in triplicate. The PCR product was extracted from 2% agarose gel and purified using the AxyPrep DNA Gel Extraction Kit (Axygen Biosciences, Union City, CA, USA), according to the manufacturer’s instructions, and quantified using a Quantus Fluorometer (Promega, Madison, WI, USA).

#### 2.5.2. Illumina MiSeq Sequencing

The purified amplicons were pooled in equimolar and paired-end sequenced on an Illumina MiSeq PE300 platform (Illumina, San Diego, CA, USA), according to the standard protocols, by Majorbio Bio-Pharm Technology Co., Ltd. (Shanghai, China). The raw reads were deposited into the NCBI Sequence Read Archive (SRA) database (Accession Number: SRP388399).

#### 2.5.3. Biodiversity Analysis

The raw reads were quality-filtered by fastp (version 0.19.6) [28] and merged by FLASH (version 1.2.11) [29] with the following criteria: (i) The reads were truncated at any site receiving an average quality score of <20 over a 50 bp sliding window, and the truncated reads shorter than 50 bp were discarded; the reads containing ambiguous characters were also discarded. (ii) The overlapping sequences longer than 10 bp were assembled according to their overlapped sequence. The maximum mismatch ratio of the overlap region is 0.2. (iii) The samples were distinguished according to their barcode and primers, and the sequence direction was adjusted, exact barcode matched, and the maximum primer mismatch number was 2.

The operational taxonomic units (OTUs) with a 97% similarity cut-off [30,31] were clustered using UPARSE version 7.1 [30], and the chimeric sequences were identified and removed. The taxonomy of each gene sequence was analyzed by the RDP Classifier version 2.2 [32] against the 16S rRNA database (Silva 138) using a confidence threshold of 0.7.

The microbial community richness and diversity analysis were analyzed utilizing Mothur (https://www.mothur.org/wiki/Download_mothur, accessed on 6 December 2021, version 1.30.2). In this study, the main measurement indices of the alpha diversity analysis were as follows: Chao1, Shannon, Ace, Simpson and Coverage indices. The taxonomic abundance analysis and beta diversity distance matrix calculation were performed with Qlime. The community species composition of each sample was calculated by the taxonomic abundance of five levels of phylum, class, order, family and genus. The hierarchical clustering of the samples was completed using the UPGMA, according to the beta diversity distance matrix [33], which could further study the relationship between the similarity or difference in the community structure of the different samples. The rarefaction curve was mainly constructed by using the microbial alpha diversity index of each sample at different sequencing depths. PLS-DA, partial least squares-discriminant analysis, can effectively distinguish the observed values between groups. LEfSe is a software tool for discovering high-dimensional biomarkers and revealing genomic features. The other analyses of the intestinal microbiota were conducted using R package software [34].

### 2.6. Statistical Analysis

The statistical analysis was performed using IBM SPSS Statistics (Version 26.0). All the data are expressed as the mean ± standard deviation. A one-way ANOVA was applied for the homogenous and normally distributed data. Otherwise, the Wilcoxon Rank-Sum Test was used. Tukey’s test was used for the multiple comparisons. *p* < 0.05 was considered a significant difference.

## 3. Results

### 3.1. Immunity

**Figure 1:**
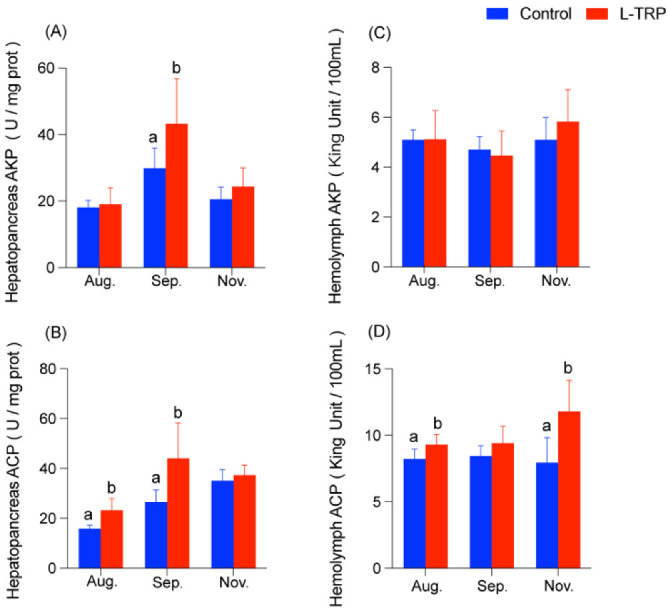
Effects of L-TRP supplementation on the AKP and ACP activities in hepatopancreas (**A**,**B**) and hemolymph (**C**,**D**) of *E. sinensis* at different sampling points (August, September and November). AKP: alkaline phosphatase; ACP: acid phosphatase. The values are expressed as the means ± SD (N = 8). Different letters above the column indicate the significant differences between the control and L-TRP groups (*p* < 0.05).

According to the results, the L-TRP supplementation had no significant effect on the AKP activity during the initial feeding period (August, Figure 1A,C). With the extension of feeding time, the AKP activity in the hepatopancreas was significantly increased in September (*p* = 0.048), but no significant difference was noted in November (Figure 1A). However, there was no significant change in the AKP activity in the hemolymph. In addition, the ACP activity in the hepatopancreas was significantly increased in the L-TRP group compared to the control group in August and September (*p* = 0.016; *p* = 0.041, Figure 1B), but no significant difference was seen in November. At the same time, the results also showed that the ACP activity in the hemolymph, after feeding the L-TRP diet, was significantly higher than in the control group in August and November (*p* = 0.017, Figure 1D).

### 3.2. Antioxidant Capacity

**Figure 2:**
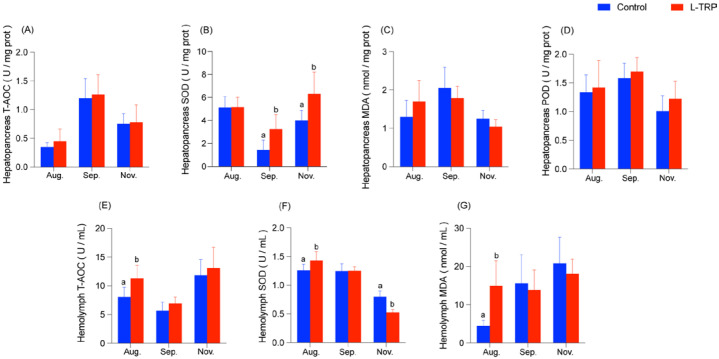
Effects of dietary L-TRP on antioxidant capacity (including T-AOC, SOD and POD) of hepatopancreas (**A**–**D**) and hemolymph (**E**–**G**) in *E. sinensis*. T-AOC: total antioxidant capacity; SOD: superoxide dismutase; MDA: malondialdehyde; POD: peroxidase. The values are expressed as the means ± SD (N = 8). Different letters above the column indicate the significant differences between the control and L-TRP groups (*p* < 0.05).

The effects of dietary L-TRP on the antioxidant capacity of *E. sinensis* are shown in Figure 2. The SOD activity in the hepatopancreas of the crabs showed no significant difference between the control and L-TRP groups in August (Figure 2B). However, the SOD activity in the hepatopancreas was significantly increased in September and November after feeding the L-TRP diet (*p* = 0.010; *p* = 0.018, Figure 2B). From August to November, the AOC, POD and MDA activities in the hepatopancreas had no significant differences between the two groups (Figure 2A–D).

As for the hemolymph, the T-AOC, SOD and MDA activities in the L-TRP group were significantly increased in August (*p* = 0.049; *p* = 0.029; *p* = 0.014, Figure 2E–G). From our results, feeding the L-TRP diet had no significant effect on the T-AOC activity in September and November (Figure 2E). In addition, the L-TRP supplementation had no significant effect on the SOD activity in September, but significantly inhibited the SOD activity in November (*p* = 0.00002, Figure 2F). In addition, there was a tendency of a decrease in the MDA activity in both September and November in the L-TRP group compared to the control group, but without a significant difference (Figure 2G).

### 3.3. Intestinal Microbiota

After the sequencing, any sample that deviated too much from the other samples was removed from each group. Therefore, there was a total of 30 samples used for further analysis (*N* = 5). According to the results, the high-throughput sequencing generated a total of 1,400,553 clean reads obtained from the 30 samples, with an average sequence length of 426 bp. According to the QIIME database, the obtained gene sequences were classified by phylum, class, order, family and genus through QIIME and on a 97% species similarity.

**Figure 3:**
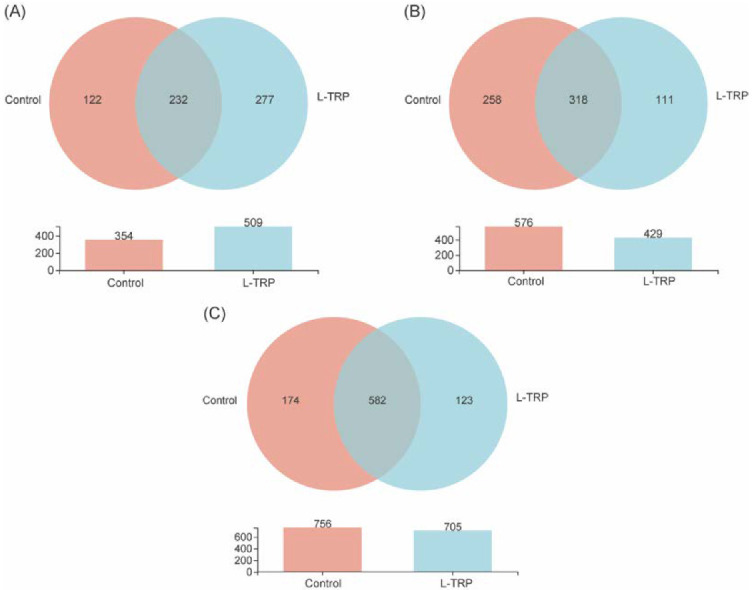
Venn diagram of the unique and shared operational taxonomic units (OTUs) in the control group and the L-TRP group in August (**A**), September (**B**) and November (**C**). The number indicates the related OTU in each group of the total sequence.

**Figure 4:**
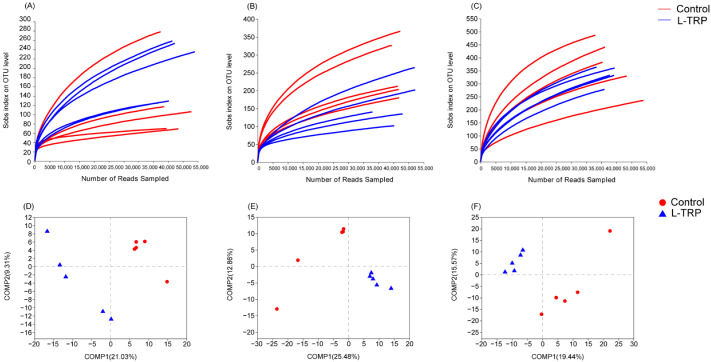
(**A**–**C**) Rarefaction curves mainly used the microbial alpha diversity index of each sample at different sequencing depths to construct the curve, which reflects the microbial diversity of each sample at different sequencing numbers in August, September and November; (**D**–**F**) PLS-DA discriminant analysis chart between the control group and L-TRP groups. The farther the distance between the different color points, the greater the difference in microbial composition between the two groups.

**Figure 5:**
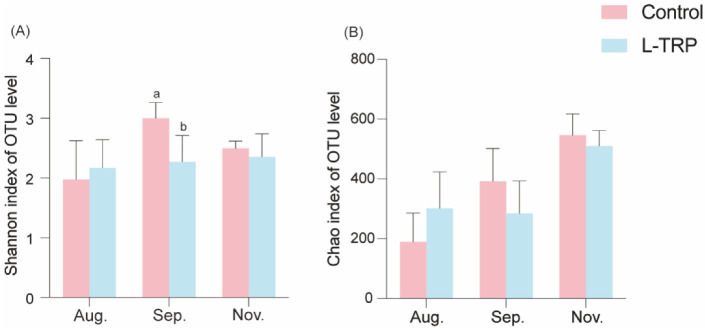
Alpha diversity analysis of biodiversity: Shannon (**A**) and Chao1 (**B**) richness and diversity index of each group (n = 5). Different letters above the column represent the significant differences between the control and L-TRP groups (*p* < 0.05).

As shown in Figure 3, in August, there were 277 OTUs in the L-TRP group and 122 OTUs in the control group, with 232 OTUs in both groups (Figure 3A). In September, there were 111 OTUs in the L-TRP group and 258 OTUs in the control group, with 318 OTUs in both groups (Figure 3B). In November, there were 123 OTUs in the L-TRP group and 174 OTUs in the control group, with a total of 582 OTUs in both groups (Figure 3C). The detailed OTUs figures and tables are uploaded to the Supplementary Materials. The coverage of each group was more than 99%. Meanwhile, the rarefaction curve shows that the curve gradually flattens out as the number of sequences increases (Figure 4A–C). This result indicates that the amount of sequencing data is large enough to reflect the diversity of the species and support the subsequent analysis. Alpha diversity indices (including Sobs, Chao1, Shannon, Ace and Simpon) were used to compare the intestinal bacterial diversity and richness of the *E. sinensis* between the control and L-TRP groups in August, September and November, respectively. Overall, the intestinal bacterial diversity (Shannon and Simpon) decreased substantially in September after feeding the L-TRP diet (*p* = 0.022; *p* = 0.037, Figure 5A). Meanwhile, the bacterial richness (Sobs and Chao1) was slightly decreased (*p* = 0.095; *p* = 0.210, Figure 5B). However, the L-TRP supplementation had no significant effect on the alpha diversity in August and November. The PLS-DA results showed that the distance of sample points is large between the control and the L-TRP groups. This result means that feeding the L-TRP diet affected the structure and species abundance of the intestinal microbiota.

**Figure 6:**
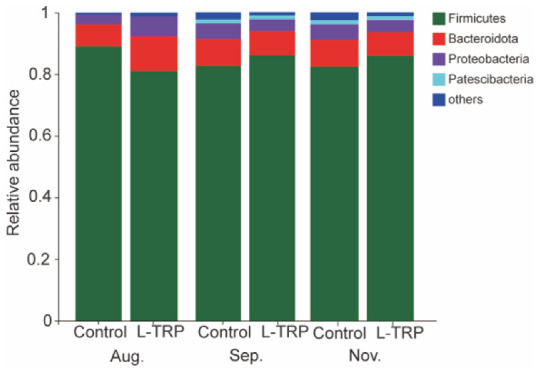
Structure and composition of the intestinal microbial composition of *E. sinensis* (phylum level) in August, September and November. Each color bar represented a phylum, and the height of the bar represents the relative abundance of each phylum, from the bottom to the top of the column are Firmicutes, Bacteroidetes, Proteobacteria, Patescibacteria and others.

**Figure 7:**
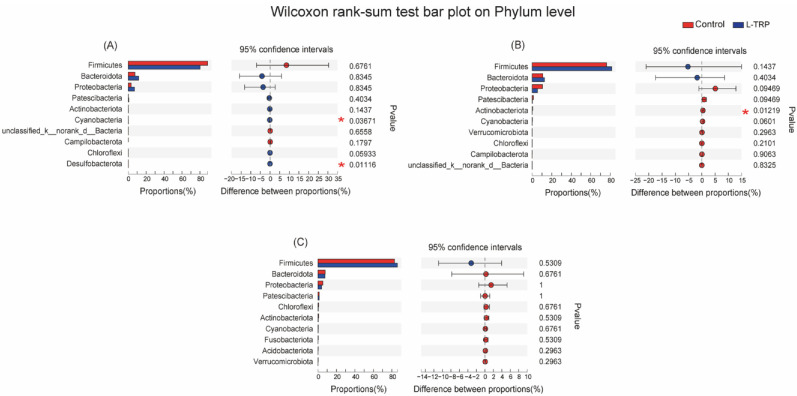
Differences in bacterial abundance in the intestinal microbial community of *E. sinensis* (phylum level) in August (**A**), September (**B**) and November (**C**). Different colored bars indicate different groups, and the rightmost value was the *p*-value that showed bacterial abundance differences between the control and L-TRP groups, * 0.01 < *p* ≤ 0.05.

**Figure 8:**
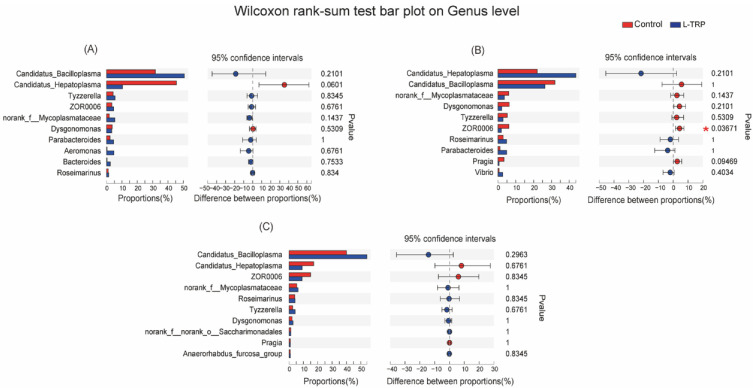
Differences in bacterial abundance in the intestinal microbial community of *E. sinensis* (genus level) in August (**A**), September (**B**) and November (**C**). Different colored bars indicate different groups, and the rightmost value was the *p*-value and showed the bacterial abundance differences between the control and L-TRP groups, * 0.01 < *p* ≤ 0.05.

In addition, our results suggested that Firmicutes, Bacteroidetes and Proteobacteria are the dominant phyla in the two groups (Figure 6). Patescibacteria was also found to be the dominant phylum in September and November (Figure 6). The results of the microbial composition and relative abundance analyses show that the dominant species at the phylum level are essentially the same for the different samples at different sampling points (Figure 7 and Figure 8). However, at the phylum level, the dietary L-TRP significantly increased the relative abundance of Cyanobacteria and Desulfobacterota in August (*p* = 0.037; *p* = 0.011, Figure 7A), and significantly decreased the relative abundance of Actinobacteriota in September (*p* = 0.012, Figure 7B). At the genus level, the L-TRP significantly decreased the relative abundance of ZOR0006 (unnamed, belonging to the Firmicutes) in September (Figure 8B).

**Figure 9:**
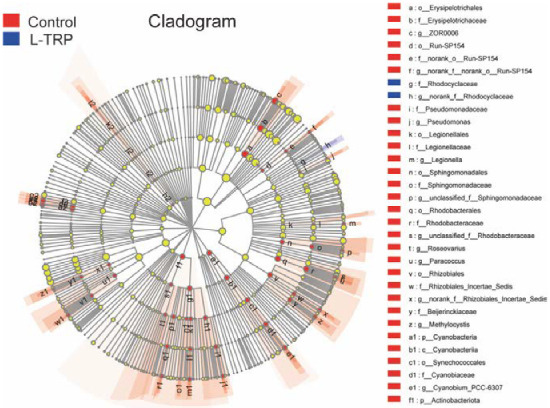
The linear discriminant analysis (LDA) effect size (LEfSe) analysis displays the differences in intestinal microbial between the control group and the L-TRP group in September. The cladogram indicates the phylogenetic distribution of microbial lineages associated with the two groups. The threshold of the logarithmic LDA score is 2.0 from the phylum-to-genus levels. Differences are represented in the color of the most abundant class (red indicates the control group; blue indicates the L-TRP group; and yellow indicates non-significant). Different letters in the figure represent different bacteria.

In the LEfSe analysis, from the phylum-to-genus levels, we analyzed the bacterial communities with significant differences between the two groups in the three months, with LDA scores over 2. A total of 40 differentially abundant taxa were identified between the control group and the L-TRP group in August. There were 31 groups of bacteria enriched in the L-TRP group, which was significantly higher than in the control group. A total of 32 differentially abundant taxa were identified in September, and *Rhodocyclaceae* (at the family level) was mainly enriched in the L-TRP group. There was no significant difference between the control group and the L-TRP group in November (Figure 9).

## 4. Discussion

According to other reports, L-TRP can affect the immune capacity of aquatic animals [12,35]. For example, our previous study reported that the short-term laboratory feeding of a L-TRP diet significantly increased the ACP and AKP activities in the serum of *E. sinensis* [35]. Machado et al. (2015) reported that dietary supplementation of tryptophan can increase the ACP activity in the plasma of *Dicentrarchus labrax* [36]. A similar result was found in young grass carp (*Ctenopharyngodon idella*) [37]. In this study, we also found that the activities of AKP and ACP have an increasing trend in the hepatopancreas and hemolymph of the *E. sinensis* after feeding the L-TRP diet in long-term pond culture. In addition, L-TRP is a precursor of melatonin. Many reports have indicated that melatonin and L-TRP exert a modulatory effect on the immune system. A previous study found that the oral administration of melatonin or TRP enhances both natural and acquired immunity in old ring doves (*Streptopelia risoria*) [38]. The AKP and ACP activities of *E. sinensis* also increased after a melatonin injection [39]. Similarly, the administration of exogenous melatonin can enhance the ACP activity in *E. sinensis* [40]. Another research study has shown that the ACP activity in the melatonin group was significantly increased 24 h after injection of *A. hydrophila* into *E. sinensis* [41]. We speculated that L-TRP may act by metabolizing to melatonin. The above results suggest that dietary L-TRP has positive effects on the immune function of *E. sinensis*.

Furthermore, antioxidant capacity can be used as a reliable indicator to assess the health status of crustaceans [13]. Antioxidant enzymes can defend against free radicals and oxidative damage in crustaceans; for example, oxygen free radicals cause damage to cellular DNA, lipids and proteins [42,43]. Therefore, we mainly evaluated the activities of T-AOC, SOD, MDA and POD in the *E. sinensis* after feeding the L-TRP diet in this study. In general, to prevent lipid peroxidation by superoxide and hydroxyl radicals, SOD promotes the superoxide to be converted into hydrogen peroxide, at first [44], and then removes excess biological reactive oxygen intermediates to avoid body harm [10]. POD can also remove excess free radicals and cooperate with SOD in animals [45]. Our results show that the SOD and POD activities of the hepatopancreas in the L-TRP group increased; by contrast, the SOD in the hemolymph was significantly decreased in November. Studies have found that dietary L-TRP significantly increases the SOD activity in sea cucumbers (*Apostichopus japonicus* Selenka) and *Channa punctatus* [46]. In addition, under high stocking density, the POD activity was significantly increased after the addition of L-TRP in Senegalese sole (*Solea senegalensis*) [47]. It is associated with L-TRP metabolites such as melatonin, 3-hydroxykynurine, and 3-hydroxyanthranilic acid, which are antioxidants [48,49]. Therefore, the addition of L-TRP could have an antioxidant defense and protect *E. sinensis* from oxidative damage. In November, unlike in the hepatopancreas, the hemolymph SOD activity was significantly decreased. Our results might suggest that the antioxidant responses had tissue-specific characteristics. One study found that the hepatopancreas seemed to have a stronger ability to defend against oxidative damages than the hemolymph in *Litopenaeus vannamei* [50]. Moreover, the decrease in the SOD activity in November may be related to other metabolites of L-TRP. For instance, IDO is involved in L-TRP metabolism as a metabolic enzyme. A study found that IDO will release more radical O_2*_^−^ during the process of redox [51]. Moreover, IDO inhibitors can increase the activity of SOD and reduce the levels of ROS in adult rats [52]. This can lead to a decrease in the SOD activity and cause tissue oxidative damage.

In addition, MDA is a biomarker of lipid modification caused by lipid peroxidation and is one of the well-defined oxidation products during the oxidation process of polyunsaturated fatty acids [53]. Excessive MDA will lead to cell damage. From our results, the content of MDA in the hepatopancreas and hemolymph increased significantly in August after feeding the L-TRP diet. According to other reports, 5-HT as a metabolite of L-TRP can elevate intracellular ROS levels [54,55]. The MDA content also increased after injection with 5-HT in rats [56]. Therefore, we speculated that the excessive concentration of L-TRP may promote 5-HT concentration in the crabs and then result in certain oxidative stress. However, the MDA content in the L-TRP group decreased in September and November (Figure 2C,G). This finding agrees with studies that were conducted on *Megalobrama amblycephala* [18] and grass carp (*Ctenopharyngodon idella*) [57]. It may be due to the different concentrations of tryptophan requirements at different developmental stages of the crabs. A study has shown that the effect of TRP in diets likely depends on the species and developmental stages [58]. In addition, juvenile crabs have higher amino acid requirements than pre-adults and adult crabs [59]. Therefore, our results suggest that the proper addition of L-TRP, according to the body size of the animals, may reduce oxidative damage. More than that, we also found that L-TRP supplementation can increase the T-AOC activity in August. The same result was confirmed in juvenile blunt-snout bream (*Megalobrama amblycephala*) after feeding the L-TRP diet [18]. As we know, T-AOC reflects the ability of the body to metabolize oxygen-free radicals [60]. Therefore, these results indicate that the proper addition of L-TRP can inhibit lipid peroxidation.

Previous studies have found that Firmicutes, Proteobacteria and Bacteroidetes are the dominant microbial communities in *E. sinensis* [12,33]. In this study, the dominant bacteria at the phylum level were also the Firmicutes, Bacteroidetes and Proteobacteria. In September and November, Patescibacteria were also found to be the dominant bacteria. This may be closely related to the change in the pond environment, such as pond sediment and water microorganisms, including zooplankton and phytoplankton. In this study, the dietary L-TRP significantly increased the relative abundance of Cyanobacteria and Desulfobacterota in August, but significantly decreased the Actinobacteriota in September. Cyanobacteria are a long-evolving group of bacteria and survive through photosynthesis. Cyanobacteria are the dominant taxa in fish, shrimp, crab and razor clams [61]. However, according to a report, the low abundance of Cyanobacteria is beneficial to the growth of the host organism [62], which could improve the immunity of *Litopenaeus vannamei*. Our results suggest that supplementation of L-TRP increases the relative abundance of Cyanobacteria and may cause potential adverse effects on the intestinal health of *E. sinensis*. However, Cyanobacteria can degrade or utilize tryptophan [63], increasing its abundance. This may be due to the crabs being small and the L-TRP concentration being too high, leading to the proliferation of the Cyanobacteria for L-TRP utilization. After the crabs had grown up in November, the relative abundance of Cyanobacteria had no significant differences between the control and L-TRP groups. In addition, Desulfobacterota participates in metabolism and is considered to regulate the amino acid, acetic acid, propionic acid and butyric acid metabolism [64]. Khan et al. (2021) suggested that the reduction of Desulfobacterota will increase the chances of opportunistic pathogens increasing, resulting in intestinal epithelial rupture and interfering with the function of the intestinal barrier. Therefore, the relative abundance of Desulfobacterota was increased in the present study, and this was beneficial to the intestinal health of the *E. sinensis*. Furthermore, a study reported that Actinobacteriota can enhance the host’s defense against pathogenic invasion by forming a defense barrier in the intestinal of blunt-snout bream (*Megalobrama amblycephala*) [65]. In addition, Wu et al. (2018) also found that Actinobacteriota can produce abundant secondary metabolites, including a variety of potent antibiotics, which can inhibit the growth of intestinal pathogens [66]. The decrease in Actinobacteriota reflects that the addition of L-TRP had an adverse effect on the *E. sinensis* in September.

At the genus level, we also found that the L-TRP supplementation significantly decreased the relative abundance of the as-yet-unnamed ZOR0006 in the Firmicutes in September. Firmicutes are considered to be sensitive indicators of host immune changes [67]. On the one hand, this may be related to the duration of the dietary L-TRP application, resulting in some adverse effects. This inference is supported by other evidence that L-TRP supplementation for 90 days has a negative impact on the overall welfare status of pikeperch (*Sander lucioperca*) [21]. However, we speculated that the change of ZOR0006 may be related to the L-TRP metabolite indole. Previous research reported that L-TRP is metabolized in the intestine and produces its metabolite indole [68]. Firmicutes can metabolize L-TRP to indole [69] and were positively correlated with indole [70]. Our results might be due to differences in the ability of the Firmicutes in the intestinal microbiota to metabolize L-TRP and promote indole production [69]. Nevertheless, the relationship between the indole and intestinal microbiota in *E. sinensis* needs to be further explored. In addition, our results showed that L-TRP increased the relative abundance of *Rhodococcus* in September by the linear discriminant analysis (LDA) effect size (LEfSe) analysis. A previous study found that the significant increase of *Rhodococcus* indicates an intestinal health improvement in juvenile yellow drums (*Nibea albiflora*) [71]. Moreover, *Rhodococcus* is thought to be involved in the denitrification process [72]. Denitrification is a vital part of the nitrogen cycle that can eliminate the toxic effects of nitric acid accumulation in organisms. Therefore, dietary L-TRP can help increase the abundance of *Rhodococcus* and thus improve the intestinal health of *E. sinensis*. In addition, it is different from the laboratory culture environment. The intestinal microbiota can be significantly affected by the change in the pond culture environment. Therefore, the composition of intestinal microbiota in the crabs might be influenced by L-TRP supplementation and the outdoor pond environment in this study. The addition of L-TRP might be excessive for the crabs in August and September, resulting in adverse effects. However, as the crabs grow, in the later stage of culture, their ability to metabolize and utilize L-TRP improves; this may reduce the effect on the intestinal microbiota.

## 5. Conclusions

In this study, we found that the supplementation of L-TRP could improve the immune capacity of *E. sinensis* in August, September and November. However, although feeding L-TRP to some extent enhanced the antioxidant capacity of the hepatopancreas in September and November, the MDA activity increased in August, and the SOD activity decreased in November, causing oxidative damage in the hemolymph. Importantly, its underlying mechanism remains worthy of further investigation. Our results showed that dietary L-TRP altered the relative abundance of certain bacteria and might have had a certain negative effect on the intestinal microbiota of the *E. sinensis* in August and September. There was no significant effect on the intestinal microbiota of the *E. sinensis* after feeding the L-TRP diet in November. We suggest that the crabs may require different concentrations of tryptophan at different developmental stages. These results can provide a scientific reference for the health regulation of *E. sinensis* in future pond culture.

## Data Availability

The data presented in this study are available in the main article.

## Supplementary Materials

The following supporting information can be downloaded at: https://www.mdpi.com/article/10.3390/metabo13010001/s1, Figure S1: The distribution of unique or common species in control and L-TRP groups in August; Figure S2: The distribution of unique or common species in control and L-TRP groups in September; Figure S3: The distribution of unique or common species in control and L-TRP groups in November. Table S1: OTU species classification statistics in August; Table S2: OTU species classification statistics in September; Table S3: OTU species classification statistics in November.
