# Supplementary material for: Effects of Dietary L-TRP on Immunity, Antioxidant Capacity and Intestinal Microbiota of the Chinese Mitten Crab (Eriocheir Sinensis) in Pond Culture
Source: Metabolites. 2022 Dec 20;13(1):1. doi: 10.3390/metabo13010001 (PMC9866439; doi:10.3390/metabo13010001)
Supplement: Supplementary file 1 [file metabolites-13-00001-s001.zip › Supplementary Material.pdf]

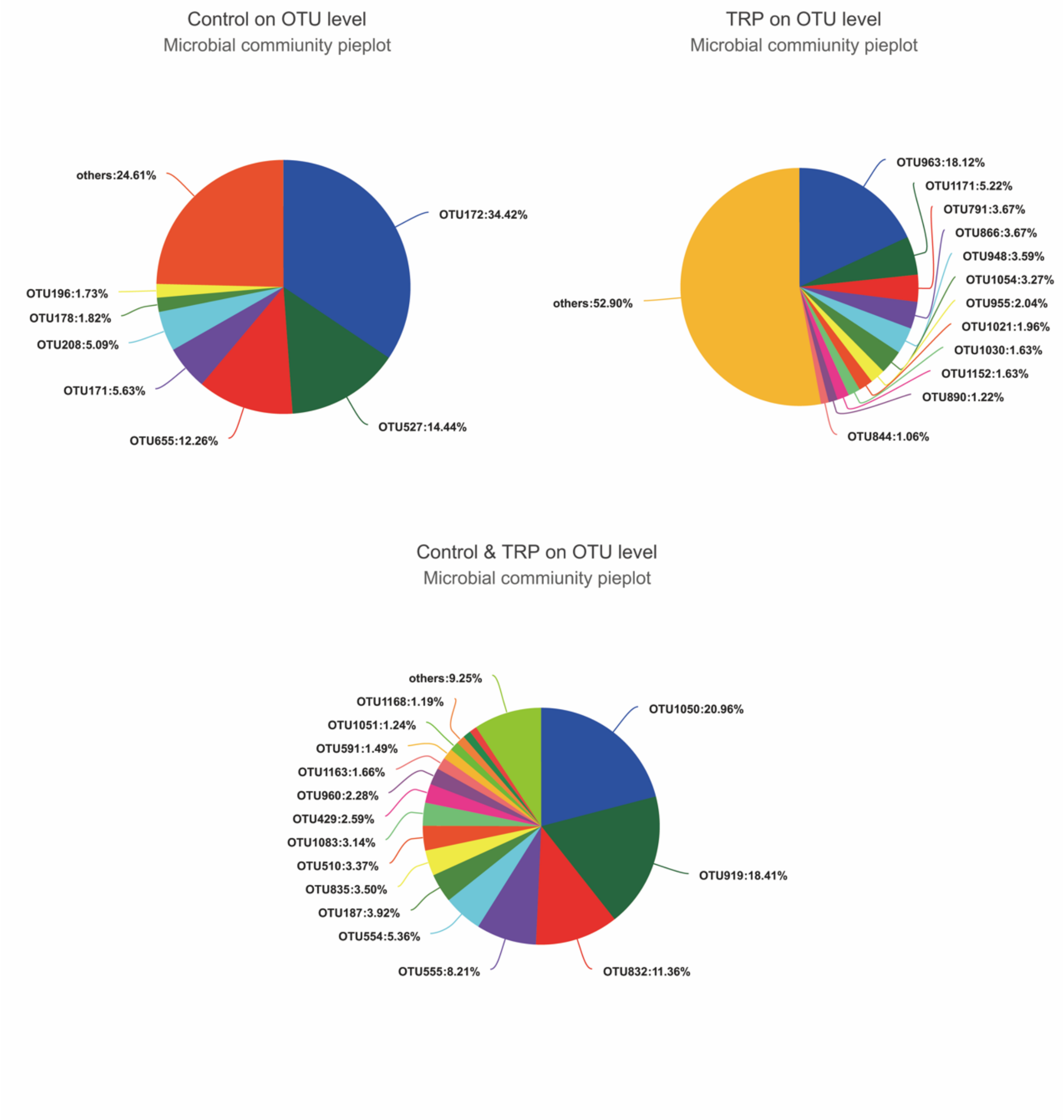

Figure S1. The distribution of unique or common species in control and L-TRP groups in August.

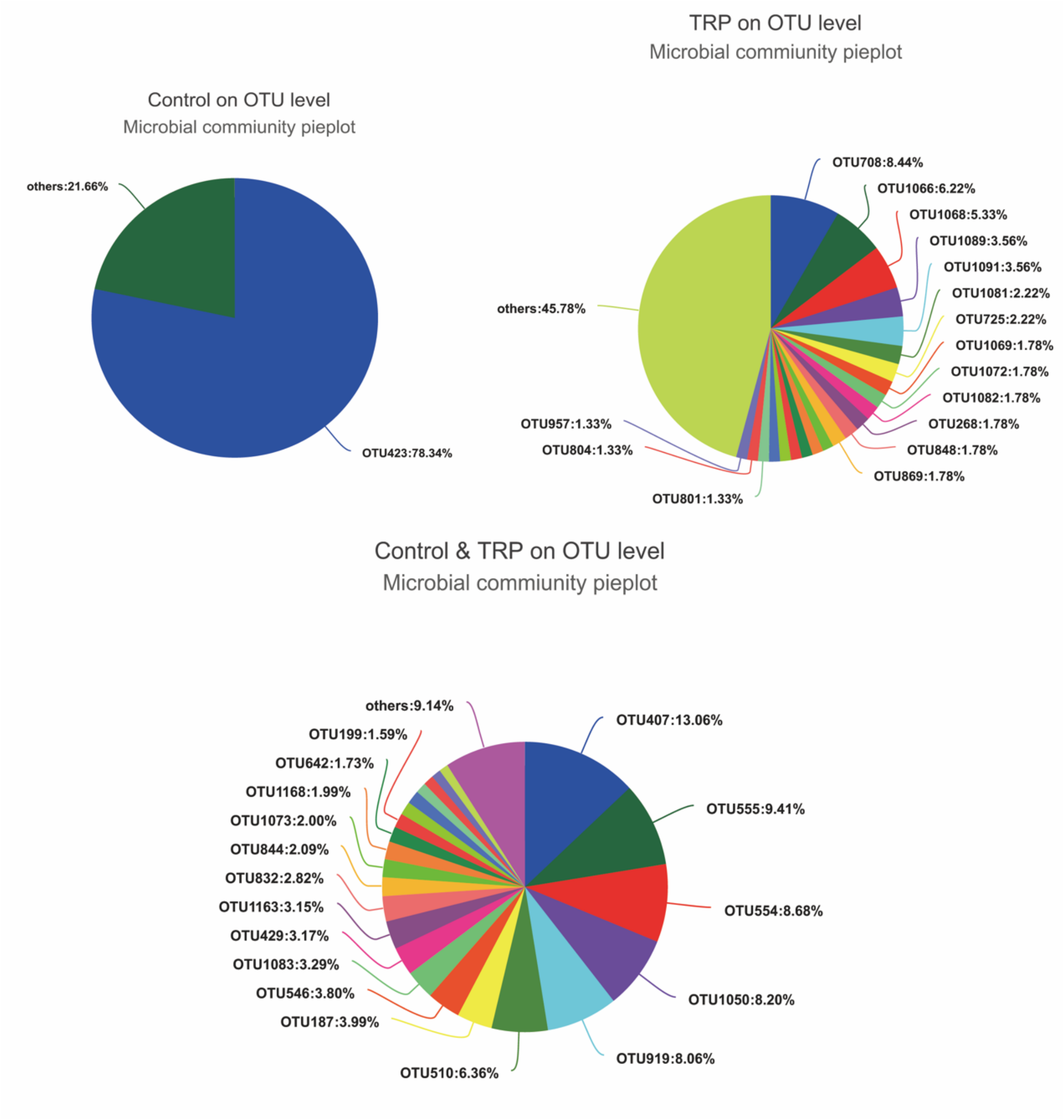

Figure S2. The distribution of unique or common species in control and L-TRP groups in September.

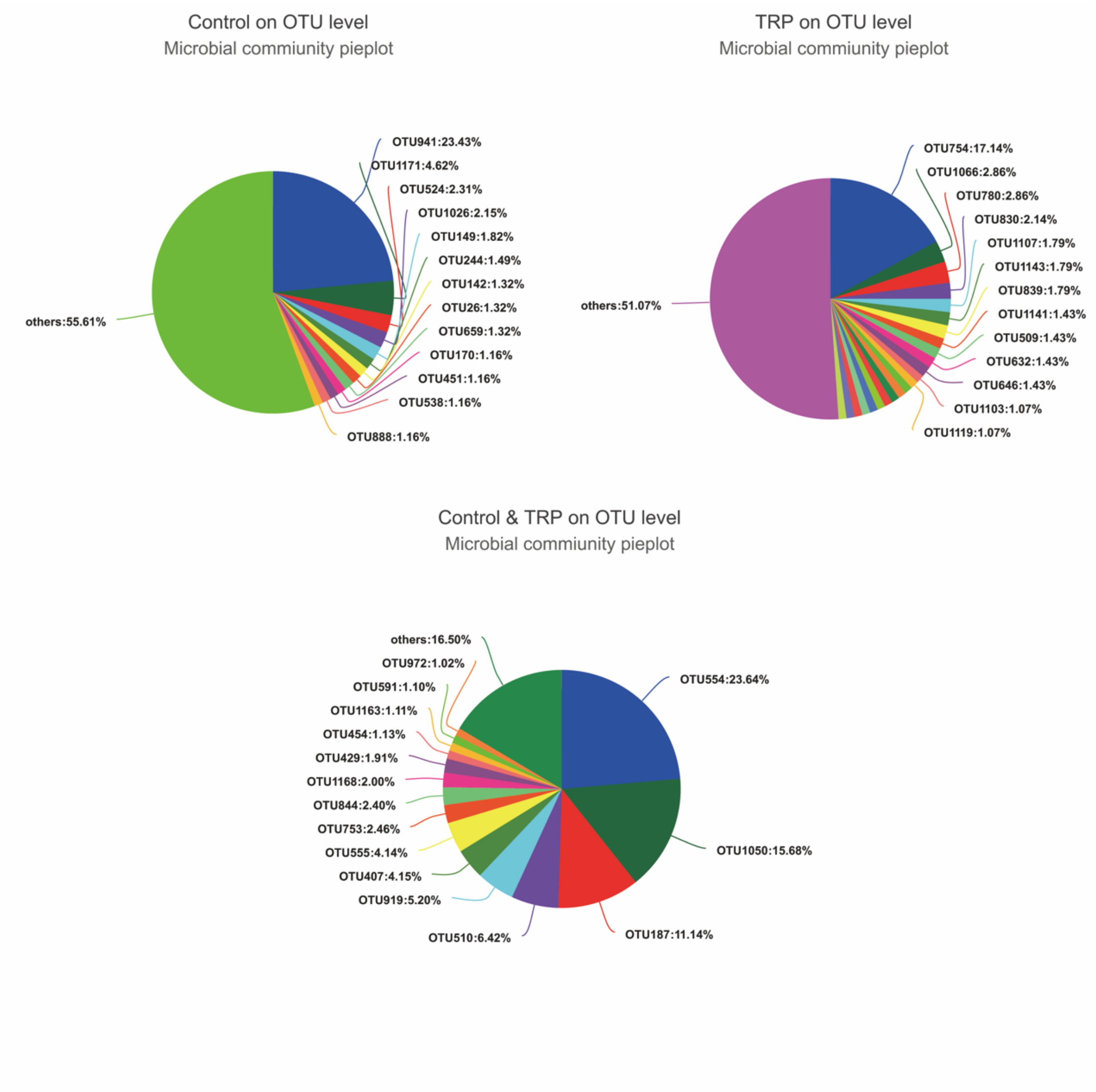

Figure S3. The distribution of unique or common species in control and L-TRP groups in November.
