# Supplementary figures and images for: Effects of Dietary L-TRP on Immunity, Antioxidant Capacity and Intestinal Microbiota of the Chinese Mitten Crab (Eriocheir Sinensis) in Pond Culture
Source: Metabolites. 2022 Dec 20;13(1):1. doi: 10.3390/metabo13010001 (PMC9866439; doi:10.3390/metabo13010001)

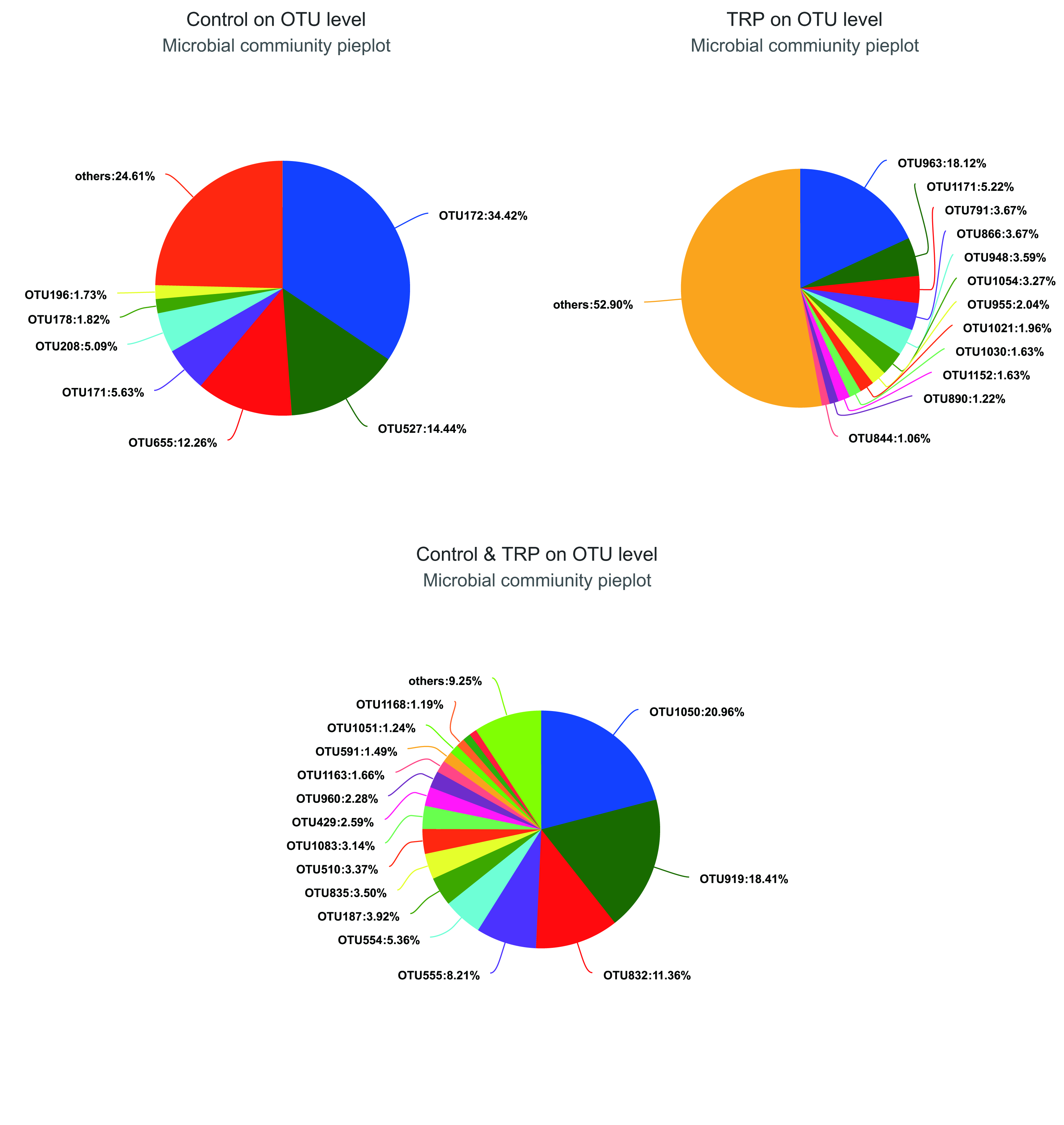

Supplement: Supplementary file 1 [file metabolites-13-00001-s001.zip › Figure S1.tif]

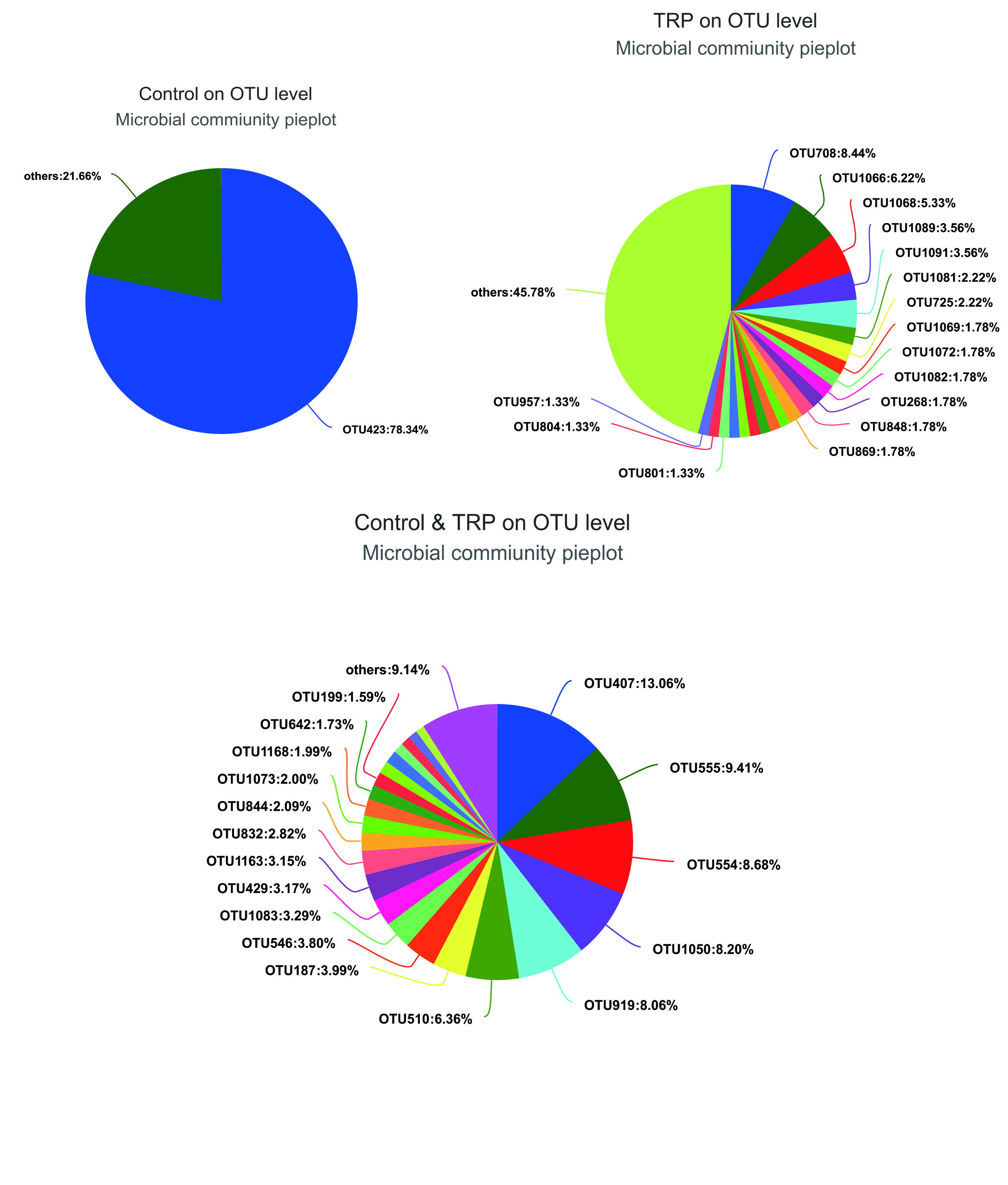

Supplement: Supplementary file 1 [file metabolites-13-00001-s001.zip › Figure S2.tif]

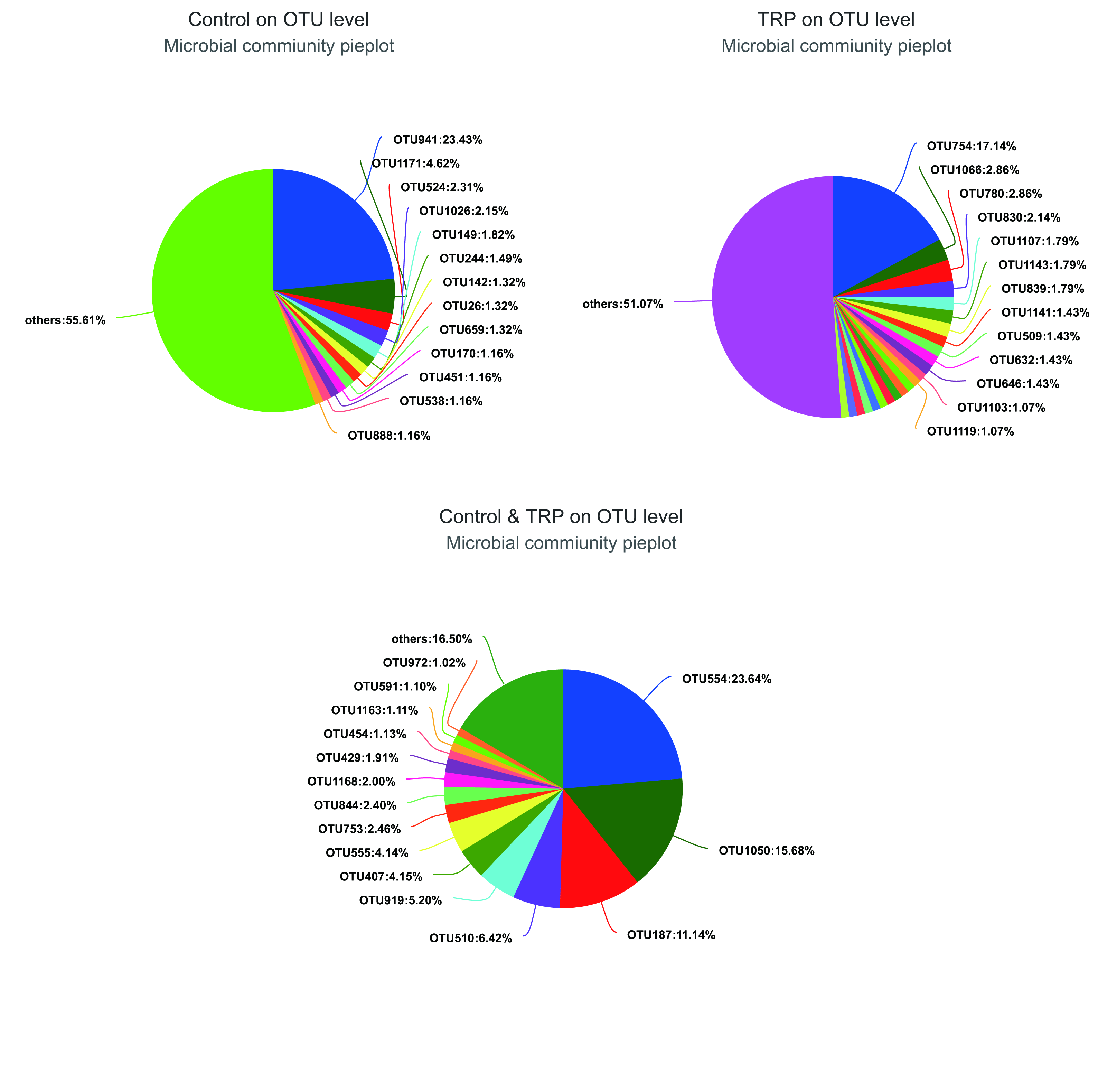

Supplement: Supplementary file 1 [file metabolites-13-00001-s001.zip › Figure S3.tif]
